# RNAseq reveals different transcriptomic responses to GA_3_ in early and midseason varieties before ripening initiation in sweet cherry fruits

**DOI:** 10.1038/s41598-021-92080-8

**Published:** 2021-06-22

**Authors:** Nathalie Kuhn, Jonathan Maldonado, Claudio Ponce, Macarena Arellano, Alson Time, Salvatore Multari, Stefan Martens, Esther Carrera, José Manuel Donoso, Boris Sagredo, Lee A. Meisel

**Affiliations:** 1 Facultad de Ciencias Agronómicas y de los Alimentos, Pontificia Universidad Católica de Valparaíso, 2340025 Valparaíso, Chile; 2 Laboratorio de Biología de Sistemas de Plantas, Departamento de Genética Molecular y Microbiología, Facultad de Ciencias Biológicas, Pontificia Universidad Católica de Chile, 8331150 Santiago, Chile; 3 Instituto de Nutrición y Tecnología de los Alimentos, Universidad de Chile, 7830490 Macul, Chile; 4 Department of Food Quality and Nutrition, Centro Ricerca e Innovazione, Fondazione Edmund Mach, Via E. Mach 1, 38010 San Michele All’Adige, Trentino Italy; 5 Instituto de Biología Molecular y Celular de Plantas, Universidad Politécnica de Valencia-Consejo Superior de Investigaciones Científicas, Ingeniero Fausto Elio s/n, 46022 Valencia, Spain; 6 Instituto de Investigaciones Agropecuarias, INIA Rayentué, Av. Salamanca S/N Sector Los Choapinos, 2940000 Rengo, Chile; 7 Programa de Doctorado en Ciencias Silvoagropecuarias y Veterinarias, Campus Sur Universidad de Chile, 820808 La Pintana, Santiago Chile

**Keywords:** Developmental biology, Genetics, Molecular biology, Plant sciences

## Abstract

Gibberellin (GA) negatively affects color evolution and other ripening-related processes in non-climacteric fruits. The bioactive GA, gibberellic acid (GA_3_), is commonly applied at the light green-to-straw yellow transition to increase firmness and delay ripening in sweet cherry (*Prunus avium* L.), though causing different effects depending on the variety. Recently, we reported that GA_3_ delayed the IAD parameter (a ripening index) in a mid-season variety, whereas GA_3_ did not delay IAD but reduced it at ripeness in an early-season variety. To further explore this contrasting behavior between varieties, we analyzed the transcriptomic responses to GA_3_ applied on two sweet cherry varieties with different maturity time phenotypes. At harvest, GA_3_ produced fruits with less color in both varieties. Similar to our previous report, GA_3_ delayed fruit color initiation and IAD only in the mid-season variety and reduced IAD at harvest only in the early-season variety. RNA-seq analysis of control- and GA_3_-treated fruits revealed that ripening-related categories were overrepresented in the early-season variety, including ‘photosynthesis’ and ‘auxin response’. GA_3_ also changed the expression of carotenoid and abscisic acid (ABA) biosynthetic genes in this variety. In contrast, overrepresented categories in the mid-season variety were mainly related to metabolic processes. In this variety, some *PP2Cs* putative genes were positively regulated by GA_3_, which are negative regulators of ABA responses, and *MYB44-*like genes (putative repressors of *PP2Cs* expression) were downregulated. These results show that GA_3_ differentially modulates the transcriptome at the onset of ripening in a variety-dependent manner and suggest that GA_3_ impairs ripening through the modification of ripening associated gene expression only in the early-season variety; whereas in the mid-season variety, control of the ripening timing may occur through the *PP2C* gene expression regulation. This work contributes to the understanding of the role of GA in non-climacteric fruit ripening.

## Introduction

Fruit ripening is a complex process that involves changes in the cell wall composition, accumulation of sugars and pigments in the peel (exocarp), cell enlargement, and the decrease in organic acid content. In non-climacteric fruits, which do not depend on an ethylene rise for ripening initiation, many hormones control this process, with abscisic acid (ABA) being the most important^1,2^. Exogenous application of ABA triggers the ripening process in non-climacteric species such as grapevine^3^ (*Vitis vinifera*), strawberry^2^ (*Fragaria spp.*), and sweet cherry^4^ (*Prunus avium*). Other hormones are involved in this process, but their participation is poorly characterized. ABA’s most characteristic feature is its sharp increase a few days before the anthocyanin accumulation^3,5^. However, ABA is not the only hormone that is present during this period. In sweet cherry, auxin, gibberellin (GAs), and cytokinins are present^5^. These hormones may modulate the ripening process together with ABA. For example, underripe grapevine berries have higher seed content and higher auxin levels in *veraison* bunches^6^, and NAA (α-naphthalene acetic acid) application delays ripening in this non-climacteric species^7^. In sweet cherry, indole-3-acetic acid (IAA) downregulates the ABA signaling pathway genes^8^.

Regarding GA, mainly GA_4_ is detected after the grapevine berry set^9^. In strawberry, GA_4_ decreases from the white to the red stage of the fruits^10^. In sweet cherry, GA_4_ significantly and inversely correlates with ripening parameters^5^. Taking these results together, GA possibly exerts a negative role in the ripening process. At the molecular level, some GAs oxidase genes change their expression at *veraison* compared to the previous stage in grapevine fruits^11^. GA_3_ treatment at *veraison* delays ripening and affects the transcript levels of putative *PP2Cs*, which are negative regulators of ABA signaling^12^. In sweet cherry, GA_3_ application delays ABA accumulation and reduces anthocyanin levels^13^.

Additionally, GA_3_ delays the fruit size increase, ripening, and color development when applied at the degreening stage^14,15,16^. Choi et al.^14^ reported that the mid-season varieties had a delayed increase in fruit size as well as polygalacturonase and Cx-cellulase activities after the GA_3_ treatment, whereas GA_3_ did not affect fruit growth in the early-season varieties. Kuhn et al.^17^ reported that the Index of Absorbance Difference (IAD), a ripening index, was delayed after the GA_3_ treatment only in a mid-season variety, whereas the treatment reduced the IAD only at harvest in an early-season variety; interestingly, both varieties presented modified expression of ABA pathway-related genes. Despite the available information on the participation of GA in the fruit ripening process, the effect of this hormone has been barely explored at the whole gene expression level during the sweet cherry fruit ripening.

There are a few transcriptomic analyses in sweet cherry. These works explore the transcriptomic features underlying fruit development^18^, fruit coloring^19^, and light-dependent anthocyanin accumulation in sweet cherry fruits^20^. Guo and co-workers^20^ found that some ABA and GA pathway genes co-expressed with light-regulated genes and suggested that both hormones may play crucial roles in light-dependent anthocyanin biosynthesis. Therefore, further exploration of the interplay of GA with ABA and other hormones during the ripening process of non-climacteric fruits is required. In grapevine, some transcriptomic studies of the effect of GA_3_ treatment during the initial fruit development have been performed^21^. In loquat (*Eriobotrya japonica*), GA_3_ applied at bloom changed the expression of ABA and auxin biosynthesis-related genes^22^. Finally, RNA-seq performed in Chinese sour cherry (*Cerasus pseudocerasus*) identified transcriptomic changes in response to GA_3_-induced parthenocarpy^23^. These studies focus on initial fruit development in which the GA participation is quite clear; however, GA’s role in the ripening process at the transcriptomic level has not been addressed to date.

Here we analyze the effect of GA_3_ at the physiological and global gene expression level in early- and mid-season sweet cherry varieties. This work aims to contribute to the understanding of the participation of GA in the fruit ripening process in non-climacteric fruits.

## Results

**Figure 1:**
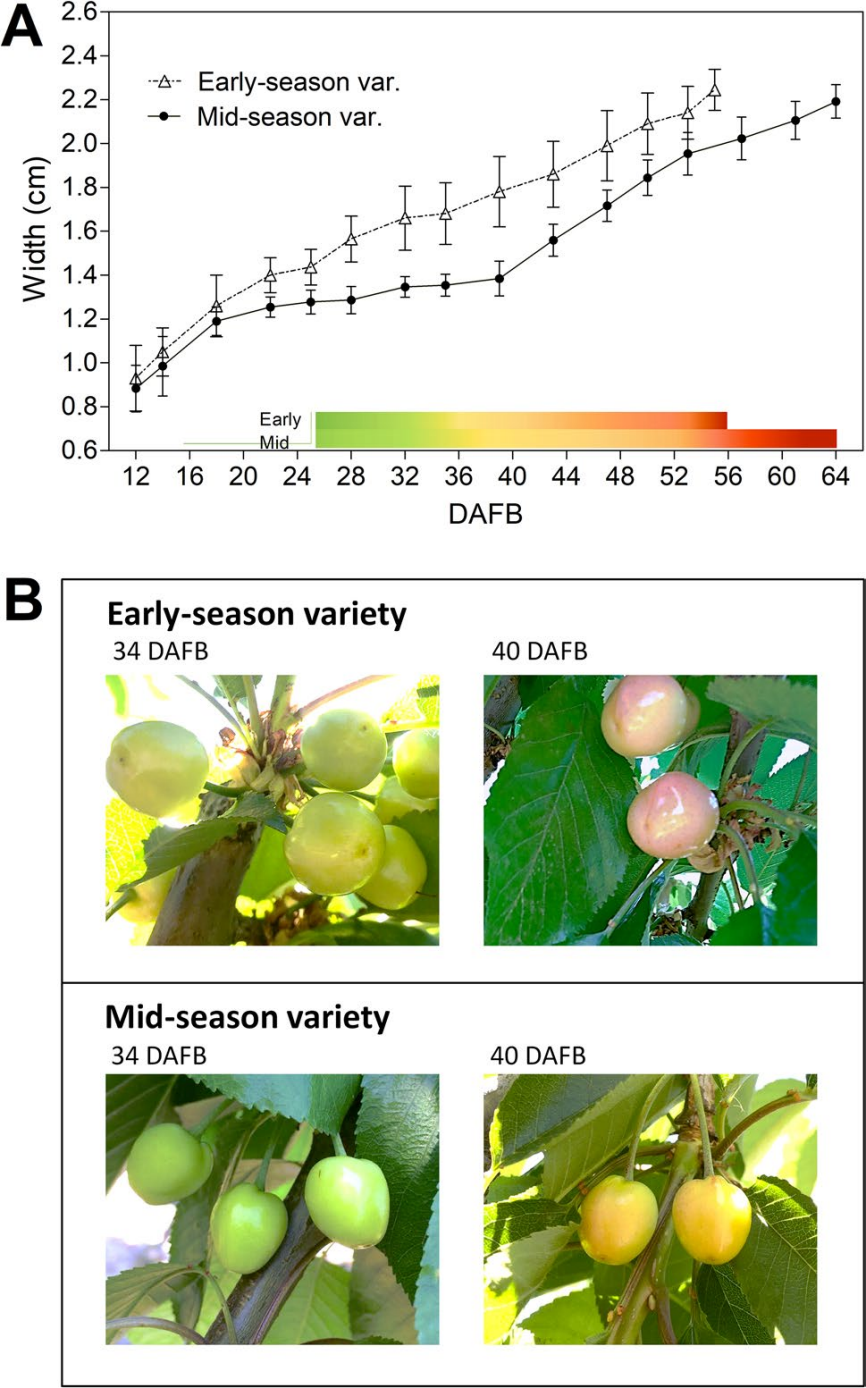
Changes in fruit size and pink color initiation in early- and mid-season varieties in the 2017–2018 season. (**A**) Fruit size as the equatorial diameter of the fruits at different days after full bloom (DAFB). Bars represent the mean of three independent biological replicates ± SD. A representation of fruit color changes is included based on the phenology of the 2017 season (Tables S1 and S2). (**B**) Representative pictures of both varieties at 34 and 40 DAFB. Graphs were performed on GraphPad Prism version 6.04 for Windows, GraphPad Software, www.graphpad.com.

‘Celeste’ and ‘Bing’ are described as early- and mid-season varieties^24^. Both varieties were characterized for fruit growth and phenology in two seasons (Fig. 1A and Fig. S1A and Tables S1 to S4). In the 2017–2018 season, the slow growth period was more prolonged in the mid-season variety, in which growth resumption occurred from 39 DAFB (Fig. 1A). On the other hand, pink color initiation started at 40 DAFB in the early-season variety (Fig. 1B), whereas in the mid-season variety, it started at 50 DAFB (Fig. 1A and Table S2). GA_4_ and GA_1_ were detected in the fruits of both varieties from 34 to 44 DAFB (Fig. S1B).

Several GAs are present at the time of fruit color initiation in sweet cherry and other non-climacteric fruits^5,10^; therefore, we aimed to investigate the role of GA in ripening triggering by altering GA homeostasis. For this, GA_3_ treatment was performed when both varieties transitioned from the light green to straw yellow fruit color (35 DAFB the 2017–2018 season and 34 DAFB the 2018–2019 season). At the time of GA_3_ application, both varieties were phenologically similar (Fig. 1; Tables S1 to S4).

GA_3_ affected ripening parameters at full ripeness in both varieties during the 2017–2018 seasons (Table 1), including firmness in both varieties, early-season variety fruit weight, and mid-season variety SSC. Similar results were observed in the 2018–2019 season (Table S5).

**Table 1:** Ripening related parameters in control- and GA_3_-treated fruit samples at harvest in early- and mid-season varieties during 2017–2018 season. d.u., durometer units; SSC, soluble solids content; M.A., malic acid.

Season	Variety	Treatment	Weight (g)	Firmness (d.u.)	SSC (ºBrix)	Acidity (M.A. %)
2017–2018	Early-season var., Celeste	Control	9.03ª**	55.63^a^	15.19^a^	2.12^a^
		GA_3_*	9.71^b^	58.33^b^	15.26^a^	1.92^a^
	Mid-season var., Bing	Control	8.70^a^	54.06^a^	19.85^a^	3.24^a^
		GA_3_	8.61^a^	63.21^b^	18.91^b^	3.19^a^

*GA_3_ was applied as the commercial product ProGibb 40% SG to individual branches at a rate of 30 ppm. GA_3_ treatment was at the light green-to-straw yellow transition of fruits at 35 DAFB in Celeste and Bing.

**For each ripening-related parameter, the significance of variation between control- and GA_3_-treated fruits was tested by one-way ANOVA analysis with Tukey's post hoc test, whereby different letters are significantly different means (*p* < 0.05).

**Figure 2:**
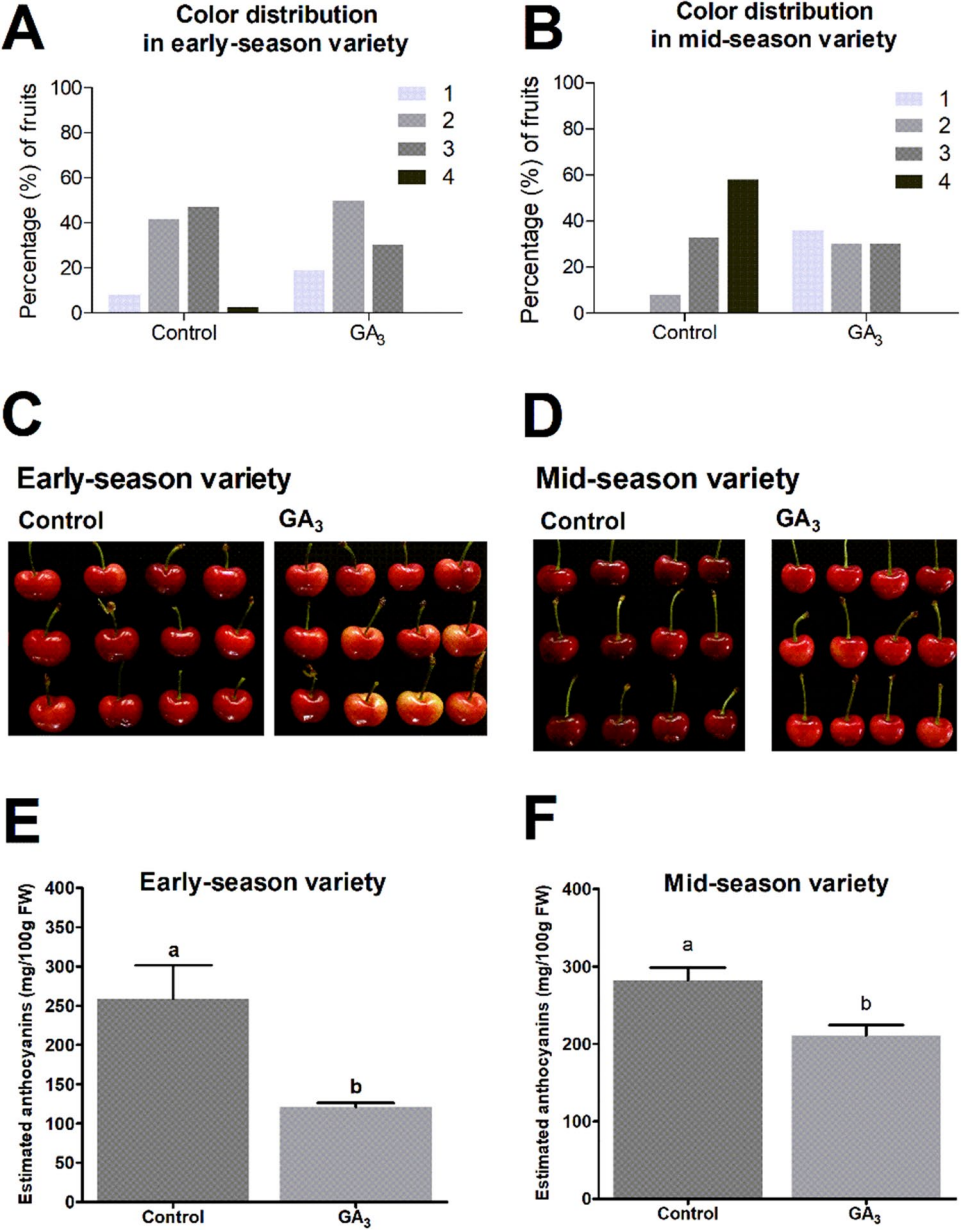
Effect of GA_3_ treatment on fruit color and estimated anthocyanin content at ripeness in early- and mid-season varieties (55 and 64 DAFB, respectively) in the 2017–2018 season. GA_3_ was applied as the commercial product ProGibb 40% SG to individual branches at a rate of 30 ppm. GA_3_ treatment was at the light green-to-straw yellow transition of the fruits at 35 DAFB in both varieties (November 2, 2017). (**A**,**B**) Color distribution according to CTIFL color chart (1 is the lightest and 4 is darkest fruit color detected). The percentage (%) is calculated considering the number of fruits having a given category over the total number of fruits. (**C**,**D**) Representative picture of control- and GA_3_-treated fruits. (**E**,**F**) Endogenous estimated anthocyanins in the fruits on a fresh weight (FW) basis, based on cyanidin-3-*O*-rutinoside content, representing more than 98% of the total anthocyanins in each of the samples. Bars represent the mean of three independent biological replicates ± SEM. The significance of variation between control- and GA_3_-treated fruits was tested by one-way ANOVA analysis with Tukey's post hoc test, whereby different letters are significantly different means (*p* < 0.05). Graphs were performed on GraphPad Prism version 6.04 for Windows, GraphPad Software, www.graphpad.com.

GA_3_ treatment produced less color in both varieties at harvest, decreased the percentage of dark fruits, and increased the percentage of light fruits in the 2017–2018 season (Fig. 2A–D), which also occurred the following season (Figs. S2A-S2D). Estimated anthocyanins were significantly reduced in both varieties at harvest (Fig. 2E,F).

**Figure 3:**
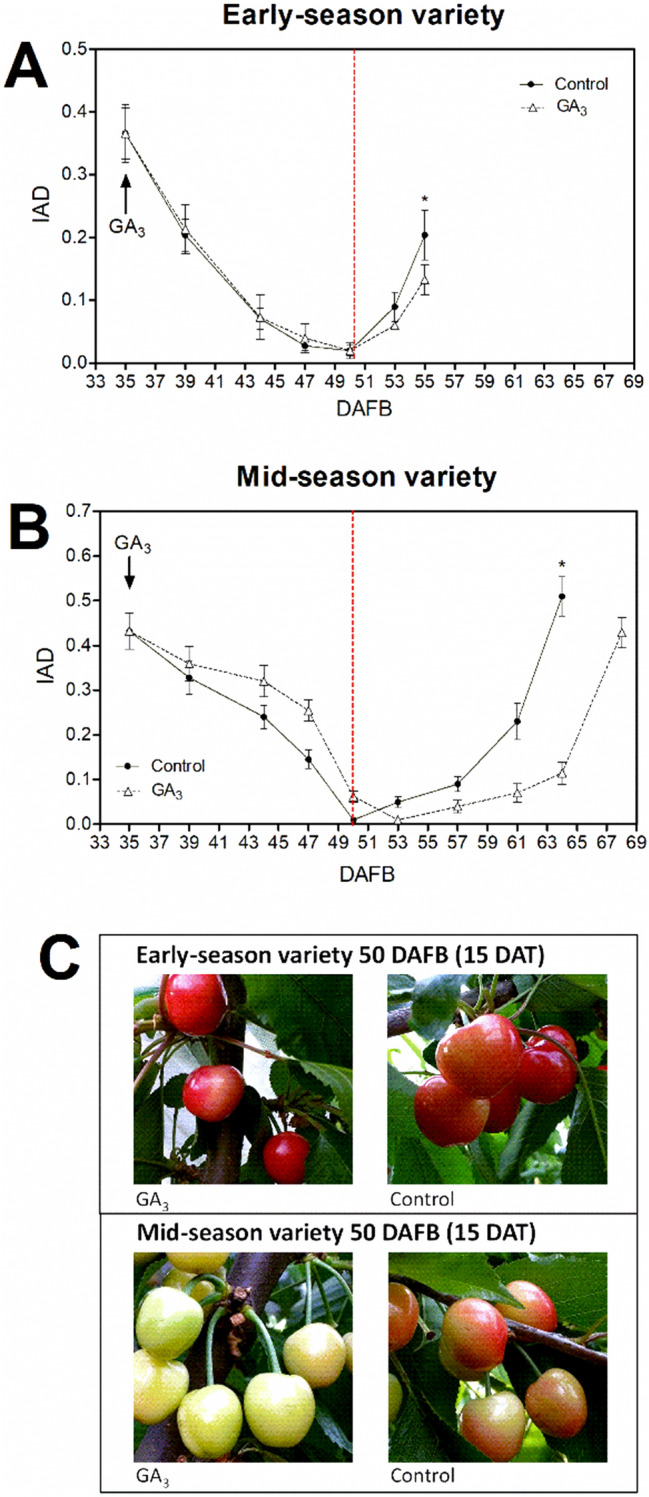
Effect of GA_3_ treatment on IAD (Index of Absorbance Difference) and fruit color in the 20172018 season. (**A**) IAD in early-season variety at different DAFB. (**B**) IAD in mid-season variety at different DAFB. GA_3_ treatment is indicated with an arrow and was performed as described in Fig. 2. In (**A**) and (**B**) bars represent the mean of three independent biological replicates ± SE. The significance of variation between control- and GA_3_-treated fruits at harvest of each variety was tested by one-way ANOVA analysis with Tukey's post hoc test, whereby an asterisk denotes significantly different means (*p* < 0.05). (**C**) Effect of GA_3_ on fruit color at 15 DAT (days after treatment). A red line indicates the moment (50 DAFB) these representative pictures were taken. Graphs were performed on GraphPad Prism version 6.04 for Windows, GraphPad Software, www.graphpad.com.

To further characterize the effect of GA_3_, IAD was measured from the date of the GA_3_ treatment in both varieties (Fig. 3).

IAD is a non-destructive ripening index utilized in sweet cherry, correlated with the anthocyanin content^25^. It estimates phenolic compounds that act as a screen for chlorophyll absorbance by measuring absorbance differences between 560 nm, 640 nm, and the 750 nm reference^26^. After the GA_3_ treatment, the early-season variety had a lower IAD value only at harvest in both seasons (Fig. 3A and Fig. S3A), whereas the mid-season variety had a delayed increase in the IAD values (Fig. 3B and Fig. S3B). This delay observed in the mid-season variety was accompanied by color differences between control- and GA_3_-treated fruits as soon as 15 days after the treatment (50 DAFB), whereas control- and GA_3_-treated fruits of the early-season variety had no differences in color at the same date (Fig. 3C).

As both varieties were differentially affected by GA_3,_ we explored the transcriptomic effect produced by GA_3_ treatment through RNA-seq analysis. For this, we analyzed fruit samples collected minutes before the GA_3_ treatment (T0) and fruit samples collected four days later (CT4 and GT4). In total, 18 samples were sequenced, nine per variety (three points: T0, CT4 and GT4, and three replicates) that yielded a total of 859 million filtered reads (84 Gb of data), with an average length of 98 bp, and a similar quantity of mapped reads to the *P. avium* transcriptome reference that was ca. 68% of the total filtered read count (Table S6).

Clustering of the 100 genes having the highest expression levels revealed that samples behaved as expected, i.e., replicates grouped, except for one replicate of T0 of early-season variety (Sample_ID: CT0_3) that was excluded from subsequent analysis (Fig. S4).

**Figure 4:**
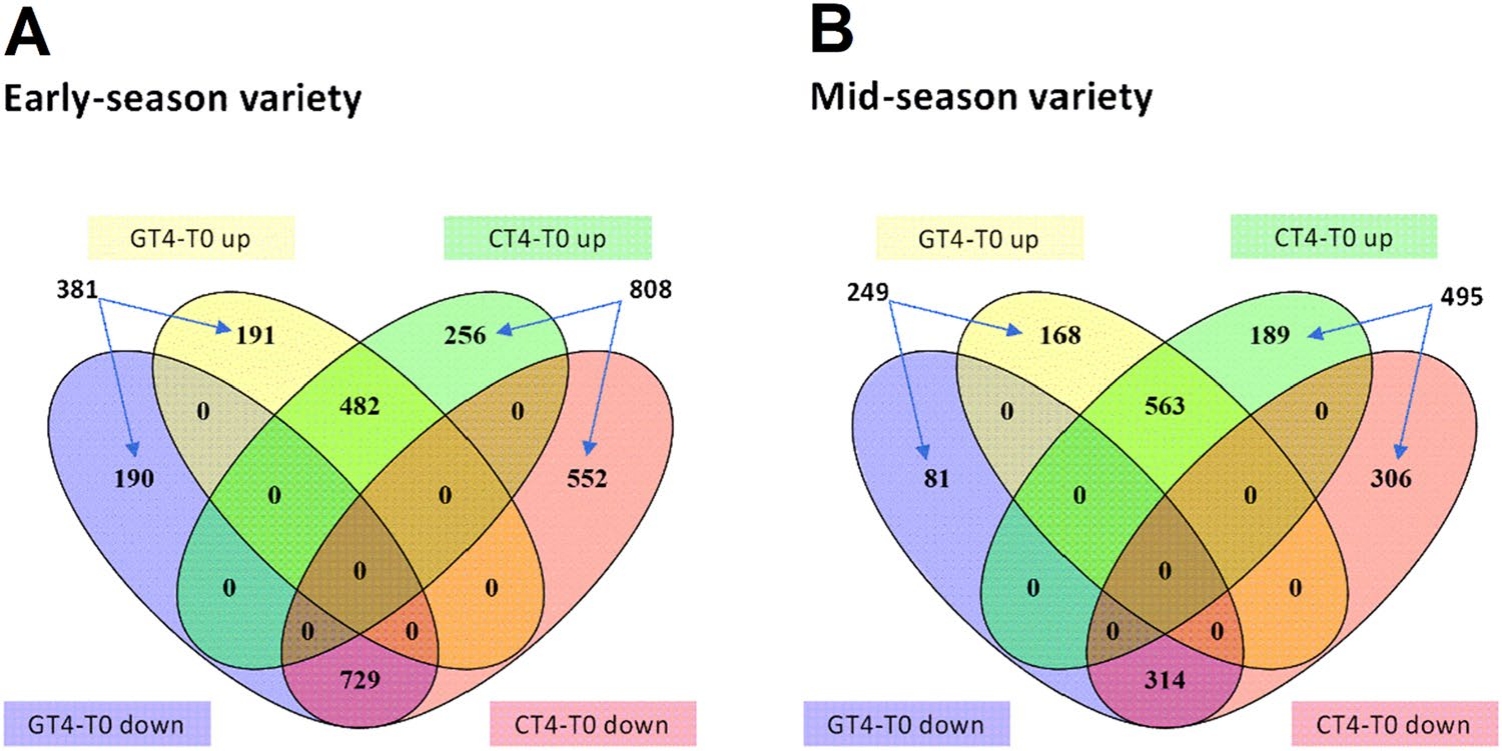
GA_3_-mediated changes in gene expression in early- and mid-season varieties in the 2017–2018 season. (**A**,**B**) Venn diagrams showing the number of differentially expressed genes (DEGs), up or downregulated, identified in the pairwise comparisons GT4-T0 and CT4-T0 of each variety, where T0 are samples collected minutes before the GA_3_ treatment and T4 are samples collected four days after the GA_3_ treatment (GT4 samples) or the control treatment (CT4 samples). DEGs were defined as having an absolute fold change value of at least two and a false discovery rate (FDR) less than < 0.05. Venn diagrams were constructed using the online tool VENNY version 2.1, http://bioinfogp.cnb.csic.es/tools/venny/index.html.

Since ripening is a process that occurs during a developmental time scale, in order to decipher the effects of GA_3_ on sweet cherry fruit ripening timing, the DEGs analysis was performed on the genes that changed between T0 and CT4 (CT4-T0) and between T0 and GT4 (GT4-T0), with a cutoff value of two-fold change and FDR < 0.05. Up and downregulated genes were identified in both varieties (Fig. 4). In the early-season variety, 381 and 808 genes changed only in GT4-T0 and CT4-T0, respectively (Fig. 4A). In the mid-season variety, 249 and 495 genes changed only in GT4-T0 and CT4-T0, respectively (Fig. 4B). There were 482 and 729 genes in the early-season variety, and 563 and 314 genes in the mid-season variety, that changed their transcript levels independently of the GA_3_ treatment (they are in the intercepts of the diagrams). In contrast, the 381 GT4-T0 genes plus the 808 CT4-T0 genes in the early-season variety, and the 249 GT4-T0 genes plus the 495 CT4-T0 genes in the mid-season variety, were GA_3_-modulated. Therefore, they were used in the subsequent GO enrichment analysis focused on the Biological Process categories.

In the early-season variety, ‘response to stimulus’, ‘response to hormone’, and ‘response to auxin’ were some of the overrepresented GO terms (FDR < 0.05) in the GT4-T0 comparison. On the contrary, ‘protein phosphorylation’ and ‘photosynthesis’ were some of the overrepresented GO terms (FDR < 0.05) in the CT4-T0 comparison (Table 2).

**Table 2:** Gene ontology (GO) enrichment analysis performed on the DEGs modulated by GA_3_ in early- and mid-season varieties. The biological process function ontologies with the lowest *p* value and FDR less than 0.05 are shown.

Variety	Comparisons	GO ID	GO description	*p*-value
Early-season var., Celeste	GT4-T0 total (381)	GO:0050896	Response to stimulus	2.284E−06
		GO:0042221	Response to chemical	6.317E−06
		GO:0010033	Response to organic substance	1.107E−05
		GO:0009725	Response to hormone	1.519E−05
		GO:0009719	Response to endogenous stimulus	1.647E−05
		GO:0009733	Response to auxin	2.071E−05
	CT4-T0 total (808)	GO:0055114	Oxidation–reduction process	9.47E−08
		GO:0006468	Protein phosphorylation	1.62E−07
		GO:0008152	Metabolic process	2.65E−07
		GO:0016310	Phosphorylation	2.09E−05
		GO:0019419	Sulfate reduction	2.57E−05
		GO:0015979	Photosynthesis	4.86E−05
Mid-season var., Bing	GT4-T0 up* (168)	GO:0005975	Carbohydrate metabolic process	5.293E−04
	CT4-T0 down* (306)	GO:0055114	Oxidation–reduction process	3.986E−08
		GO:0009607	Response to biotic stimulus	3.269E−05
		GO:0005975	Carbohydrate metabolic process	4.311E−05
		GO:0006950	Response to stress	4.565E−05

*GT4-T0 up and CT4-T0 down comparisons of mid-season variety had more overrepresented categories than GT4-T0 total and CT4-T0 total, respectively.

Regarding the mid-season variety, the GO term ‘carbohydrate metabolic process’ was overrepresented (FDR < 0.05) in the GT4-T0 comparison with upregulated genes, whereas ‘oxidation–reduction process’ and ‘response to stress’, among others, were overrepresented GO terms (FDR < 0.05) in the CT4-T0 comparison that includes downregulated genes (Table 2).

**Figure 5:**
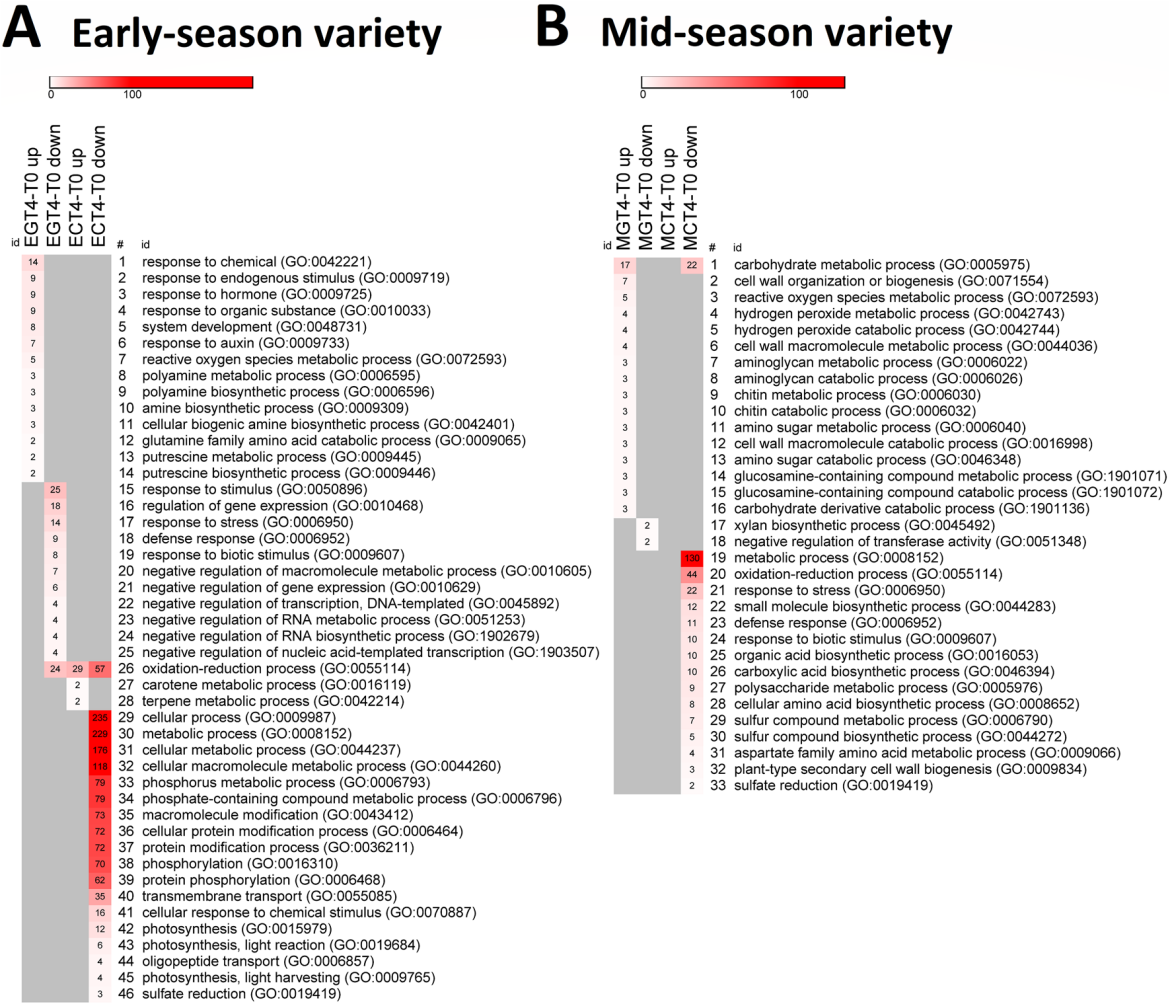
Heat maps with the number of DEGs modulated by GA_3_ in each Gene Ontology (GO) category. (**A**) Number of genes in GT4-T0 and CT4-T0 comparisons that are up or downregulated in the early-season variety. (**B**) Number of genes in GT4-T0 and CT4-T0 comparisons that are up or downregulated in the mid-season variety. For (**A**,**B**) biological processes with *p* < 0.001. Heatmaps were constructed with the online tool Morpheus, https://software.broadinstitute.org/morpheus/.

In general, GO terms were specific to a particular comparison, except for the GO category ‘oxidation–reduction process’ in the early-season variety (Fig. 5A) and ‘carbohydrate metabolic process’ in the mid-season variety (Fig. 5B), with downregulated genes in CT4-T0 and upregulated genes in the GT4-T0. The GO term ‘metabolic process’ had the highest number of DEGs modulated by GA_3_ in the mid-season variety (Fig. 5B). In the early-season variety, this GO term and ‘cellular process’ were the most abundant (Fig. 5A).

CT4-T0 up and down comparisons contain GA-modulated genes that up or downregulated from T0 to T4, but they no longer changed after the GA_3_ treatment. In the early-season variety, the CT4-T0 down comparison contained DEGs in ‘photosynthesis’, ‘protein phosphorylation’, and ‘transmembrane transport’ GO categories, among others (Fig. 5A). In contrast, mid-season variety contained DEGs in the ‘response to stress’ and ‘plant-type secondary cell wall biogenesis’ GO categories, among others (Fig. 5B). On the other hand, the ‘carotenoid metabolism’ GO category contained GA-modulated genes that upregulated from T0 to T4 in the early-season variety.

GT4-T0 up and down comparisons contain GA-modulated genes that did not change from T0 to T4 but are up or downregulated by GA_3_. DEGs in the ‘auxin response’ and ‘putrescine biosynthetic process’ GO categories were present in the GT4-T0 up comparison in the early-season variety. In contrast, several GO categories related to negative regulation of gene expression were found in the GT4-T0 down comparison in this variety (Fig. 5A). In the mid-season variety, DEGs in the ‘cell wall organization or biogenesis’ and ‘reactive oxygen species metabolic process’ GO categories were present in the comparison containing upregulated genes (GT4-T0 up), whereas ‘xylan biosynthetic process’ had DEGs in the GT4-T0 down comparison in this variety (Fig. 5A).

**Figure 6:**
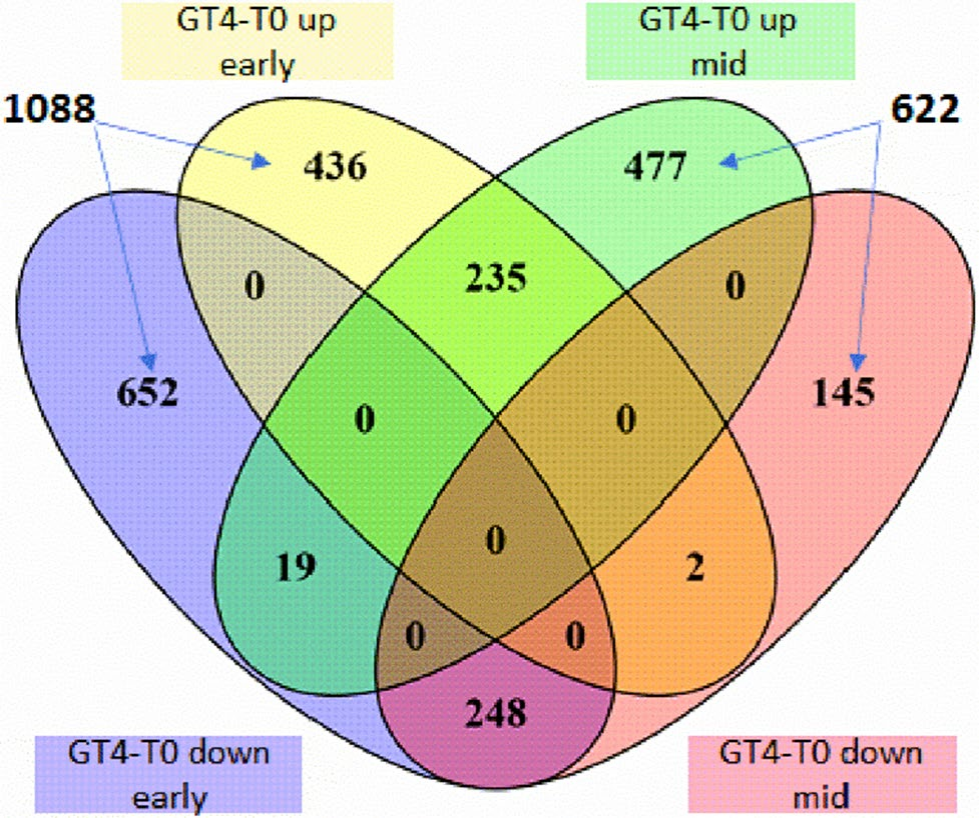
GA_3_-mediated changes unique of early- or mid-season varieties in the 2017–2018 season. Venn diagrams showing the number of DEGs, up or downregulated, identified in the pairwise comparisons GT4-T0 in both varieties. DEGs were defined as in Fig. 4. Venn diagrams was constructed using the online tool VENNY version 2.1, http://bioinfogp.cnb.csic.es/tools/venny/index.html.

After that, we investigated the variety-specific genes that respond to GA_3_ treatment (Fig. 6). In the GT4-T0 comparison of the early-season variety, 436 and 652 genes were up and downregulated, respectively. On the other hand, 477 and 145 genes were up and downregulated in the mid-season variety.

As shown in Table 3, the GO terms ‘response to auxin’, ‘response to stress’, and ‘DNA packaging’, among others, were overrepresented (FDR < 0.01), with genes that exclusively changed in the early-season variety. On the other hand, in the mid-season variety, ‘regulation of transcription, DNA-templated’ and ‘regulation of nucleic acid-templated transcription’ were some of the overrepresented GO terms (FDR < 0.01) in the upregulated DEGs that were exclusive of this variety.

**Table 3:** GO enrichment analysis performed on the DEGs modulated by GA_3_ and unique to each variety. The biological process function ontologies with the lowest *p*-value and FDR less than 0.05 are shown.

Variety	Comparisons	GO ID	GO description	*p*-value
Early-season var., Celeste	GT4-T0 total (1088)	GO:0050896	Response to stimulus	1.34E−13
		GO:0055114	Oxidation–reduction process	1.31E−11
		GO:0042221	Response to chemical	2.90E−10
		GO:0009733	Response to auxin	7.08E−10
		GO:0006950	Response to stress	6.87E−09
		GO:0006323	DNA packaging	9.67E−09
Mid-season var., Bing	GT4-T0 total (622)	GO:0006355	Regulation of transcription, DNA-templated	2.90E−06
		GO:1903506	Regulation of nucleic acid-templated transcription	3.48E−06
		GO:2001141	Regulation of RNA biosynthetic process	3.48E−06
		GO:0009409	Response to cold	4.13E−06
		GO:2000112	Regulation of cellular macromolecule biosynthetic process	5.81E−06
		GO:0051252	Regulation of RNA metabolic process	5.94E−06

**Figure 7:**
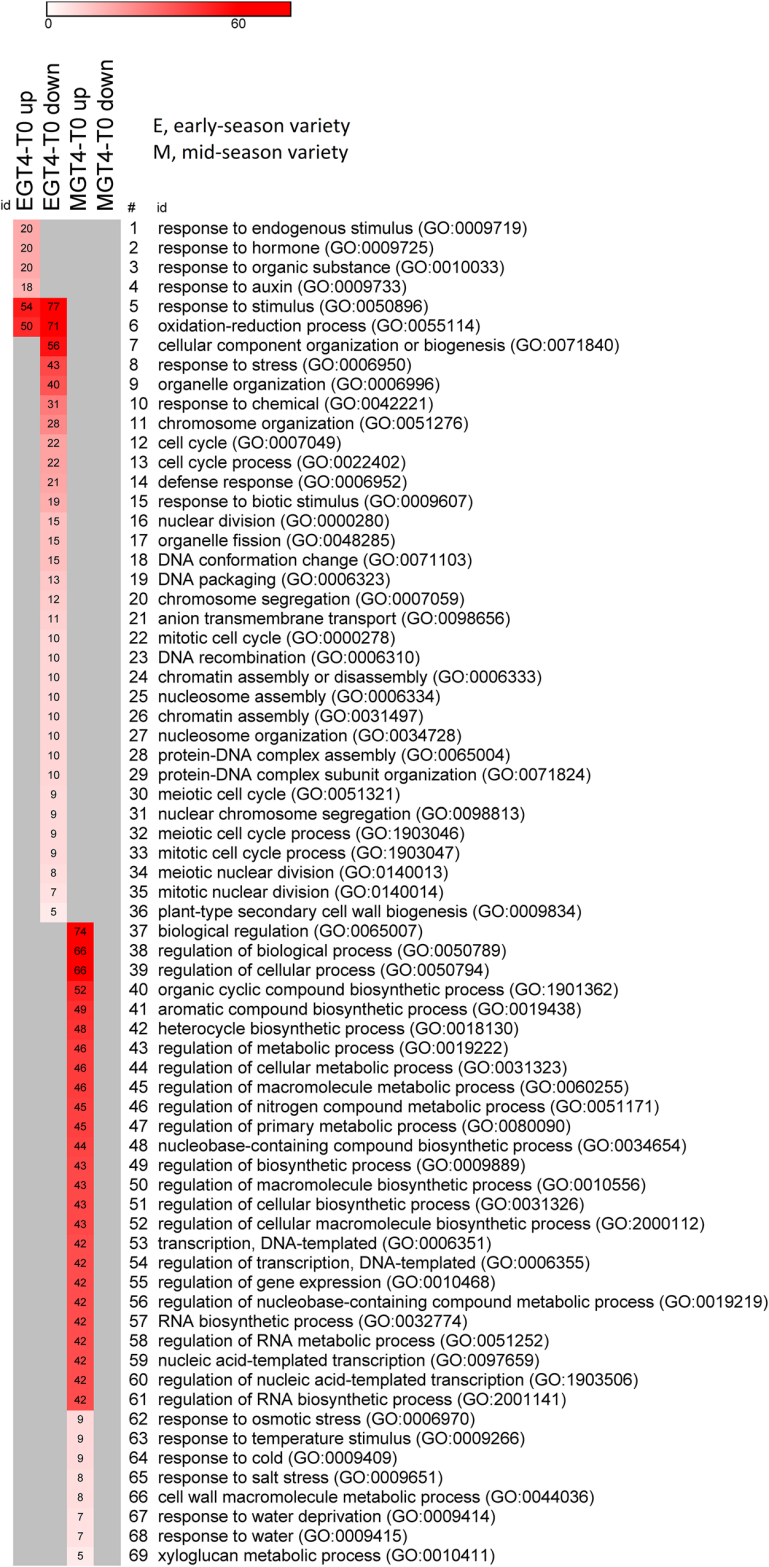
Heat map with the number of DEGs unique of each variety in each Gene Ontology (GO) category. The comparison GT4-T0 with genes up or downregulated in the early-season variety and mid-season variety Bing is presented. Biological processes with FDR < 0.05. Heatmap was constructed with the online tool Morpheus, https://software.broadinstitute.org/morpheus/.

In Fig. 7, the enriched GO terms that had more DEGs unique to the early-season variety were ‘response to stimulus’, ‘oxidation–reduction’ process’, ‘cellular component organization or biogenesis’, ‘chromosome organization’, ‘cell cycle’, ‘response to hormone’, ‘response to auxin’, among others. In contrast, the GO terms that had more DEGs unique to the mid-season variety were ‘biological regulation’, ‘aromatic compound biosynthetic process’, ‘regulation of transcription, DNA-templated’, and ‘regulation of nucleic acid-templated transcription’, among others (Fig. 7).

The differential effect of GA_3_ on the coloring initiation between varieties was accompanied by differences in the expression of genes encoding putative anthocyanidin 3-*O*-glucosyltransferase like proteins. For instance, in the early-season variety, GA_3_ reduces the increase in the transcript levels of anthocyanidin 3-*O*-glucosyltransferase 2-like gene that occurs during fruit development (CT4-T0). In contrast, in the mid-season variety, GA_3_ avoids the inhibition of the reduction in transcript levels that occur during fruit development (CT4-T0) in the anthocyanidin3-*O*-glucosyltransferase 5-like gene (Table 4). In the mid-season variety, two genes encoding putative anthocyanin reductase (ANR) increased their expression after the treatment; the two genes encoding putative leucoanthocyanidin reductase (LAR) proteins had opposite behaviors (Table 4).

**Table 4:**
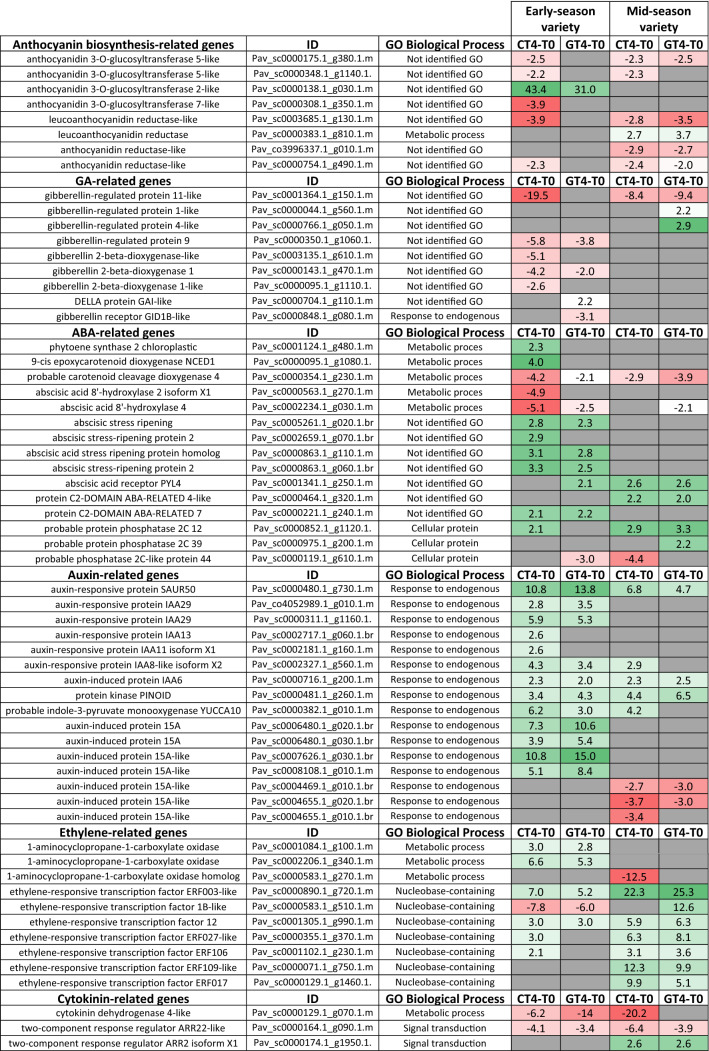
Differential gene expression as fold change of genes related to anthocyanin synthesis and hormone pathways in pairwise comparisons CT4-T0 and GT4-T0 of each variety. In gray, comparisons having genes whose absolute fold change value was at least two.

As ‘response to hormone’ was an overrepresented GO, a directed search of hormone-related genes was performed in the CT4-T0 and GT4-T0 comparisons of each variety (Table 4).

Several gibberellin 2-beta-dioxygenase related genes were downregulated in the CT4-T0 and GT4-T0 comparisons only in the early-season variety. Regarding the ABA biosynthetic pathway, two abscisic acid 8’-hydroxylase related genes were downregulated in the early-season variety. Concerning the ABA response, several abscisic stress ripening-related genes were upregulated in the CT4-T0 comparison only in the early-season variety, which was less marked in the GT4-T0 comparison. Regarding the auxin response, several genes that encode putative auxin AuxIAA response repressors were induced in the CT4-T0 comparison of the early-season variety, and they were less upregulated in the GT4-T0 comparison. Some genes coding for auxin-induced protein 15A and 15A-like were upregulated in CT4-T0 and were upregulated even more in GT4-T0, whereas other groups of auxin-induced protein 15A-like were downregulated in the mid-season variety. In contrast, several ethylene-responsive transcription factor genes were more upregulated in the mid-season variety than in the early-season variety.

Given that ‘negative regulation of gene expression’ and ‘DNA packaging’ and ‘nucleosome assembly’ GO terms were overrepresented, a directed search of genes related to these categories was performed (Table 5).

**Table 5:**
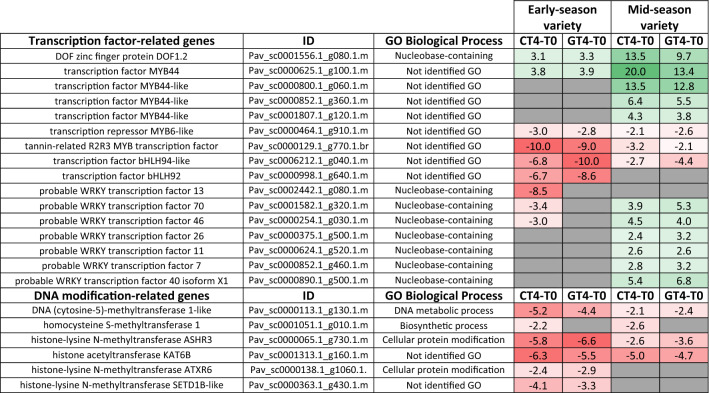
Differential gene expression as fold change of genes related to transcriptional and epigenetic regulation in pairwise comparisons CT4-T0 and GT4-T0 of each variety. In gray, comparison having genes whose absolute fold change value was at least two

Many genes coding for MYB44-like transcription factor were upregulated in CT4-T0, but they were less induced in the GT4-T0 comparison in the mid-season variety. In contrast, other transcription factor genes were downregulated in the early-season variety, including a gene coding for a putative tannin-related R2R3 MYB. Finally, both varieties had downregulated genes encoding putative histone methyltransferases and a DNA (cytosine-5)-methyltransferase 1-like in the CT4-T0, which were slightly less expressed in the GT4-T0 comparison.

## Discussion

### Physiological differences between early- and mid-season varieties

Sweet cherry varieties are considered early-, mid-, or late-season depending on the harvest time, which depends on the flowering time and the fruit developmental period^24^. In our experimental conditions, early- and mid-season varieties flowered the same day in the 2017–2018 season and within a two-day time frame in the 2018–2019 season. Therefore, the varieties utilized in this work differed mainly in their fruit developmental length and, more specifically, in the duration of the slow-growth period that occurs during the light green-to-straw yellow transition, which was longer in the mid-season variety (Fig. 1A), similar to what has been reported previously^14^. These results imply that the ripening processes, including color initiation, begin earlier in the early-season varieties. In fact, in the early-season variety, pink color initiates at 40 DAFB (Fig. 1B), whereas in the mid-season variety, it occurred from 50 DAFB onwards, several days after growth resumption (Fig. 1A).

GA_1_ and GA_4_ have been reported to be present at the onset of ripening in sweet cherry fruits, and GA_4_ negatively correlates with ripening parameters^5^. We found that the color initiation differences between both varieties were accompanied by differences in the GA_4_ and the GA_1_ content. For instance, the early-season variety, which colors first, had lower GA_4_ levels than the mid-season variety at 38 and 44 DAFB (Fig. S1B); and GA_1_ levels were higher in the mid-season variety at 38 DAFB (Fig. S1C); therefore, possibly low levels of GAs are needed for the ripening triggering. In strawberry, another non-climacteric fruit, color change coincides with the GA_4_ decrease between the white and the red stage^10^. In the non-climacteric sweet pepper (*Capsicum annuum* L.), silencing of DNA methylase gene *CaMET1-like1* caused repressed expression of GA biosynthesis genes and led to premature ripening^27^. Taking this evidence together, GA could be a negative regulator of ripening, explaining the differences in the timing of the color development initiation between contrasting sweet cherry varieties.

### Differential effect of GA_3_ on fruit quality parameters between early- and mid-season varieties

To further investigate GA’s role in ripening triggering, we altered GA homeostasis by treating fruits with GA_3_, a GA commonly used in agronomic practices. The treatment increased firmness in both varieties. The firmness effect of GA_3_ may be associated with changes in cell wall composition. Kondo and Danjo^13^ found that during sweet cherry fruit ripening, cell wall-bound neutral sugars—galactose, arabinose, among others—were reduced, which is associated with fruiting softening, and that GA_3_ treatment prevented these decrease, supporting the role of GA in fruit firmness maintenance.

It is commonly accepted that early-season varieties are unresponsive to GA_3_; this is based on some reports showing no effect of GA_3_ on some ripening-related parameters. For instance, Choi et al.^14^ found that GA_3_ did not affect fruit size and firmness in early-ripening genotypes. In contrast, we found that GA_3_ impaired color development, reduced IAD, and increased fruit weight at harvest in the two seasons evaluated (Fig. 3A and Fig. S3A; Table 1 and Table S5), similar to our recent report in other early-season variety, where IAD was lower in GA_3_-treated fruits compared with control fruits^17^. In the early-season variety Satohnishiki, GA_3_ treatment decreased anthocyanin fruit content and modified the sugar accumulation pattern^13^.

On the other hand, it is usually affirmed that GA_3_ affects ripening-related parameters in mid- or late-season varieties because of a delay in softening, fruit size increase, and soluble solids accumulation^14,15,16^. However, since the GA_3_-treated fruits ripen similarly to control fruits and only need more time to acquire the desired features, it would be correct to refer to this as a delay. The idea of a ripening impairment arises from studies that analyzed only the harvest point and found less color in the GA_3_-treated fruits^16,28^. Therefore, the monitoring of fruit-ripening parameters is crucial for a better understanding of the physiological effect of GA_3_ on late- or mid-season varieties. For this, we used the IAD ripening index^26^, already utilized in the context of sweet cherry fruit ripening^25^. We observed a color difference as soon as 15 days after the GA_3_ treatment (Fig. 3C), accompanied by an IAD delay in the GA_3_-treated fruits of the mid-season variety (Fig. 3B). This delay was also found in another mid-season variety^17^, which supports the idea that mid-season varieties respond differently to GA_3_ treatment than early-season varieties. GA_3_-treated fruits of the mid-season variety had less color, anthocyanin content, and IAD value at harvest (Fig. 2 and S2). However, the IAD value of GA_3_-treated fruits reached similar control values four or five days later (Fig. 3 and S3), suggesting no ripening impairment in the mid-season variety; rather, there is a delay in the ripening process. A previous report showed that late- and mid-season varieties had delayed color development after the GA_3_ treatment, and maturity was two to three days delayed in the GA_3_-treated fruits^16^.

At the molecular level, differences in the coloring process between varieties were accompanied by differences in the expression of genes encoding putative anthocyanidin 3-O-glucosyltransferase like proteins (Table 4), where the transcript abundance of the gene that encodes the anthocyanidin 3-O-glucosyltransferase 2-like putative protein decreased in the early-season variety, which could explain less color of this variety at harvest. As shown in Table 4, GA_3_ increased the expression of two genes encoding putative anthocyanin reductase (ANR) and one gene encoding a leucoanthocyanidin reductase (LAR) in the mid-season variety. ANR and LAR proteins redirect the anthocyanin route to flavan-3-ols, causing less anthocyanin production, and this could be related to the “delayed fruit coloring” phenotype.

### GA ripening initiation control differs between early- and mid-season varieties at the transcriptomic level

The differential physiological response to exogenous GA_3_ between early- and mid-season varieties, and the advanced color initiation in the early-season variety, which is accompanied by lower GAs levels, led us to hypothesize that the ripening process of these contrasting varieties is very different at the molecular level, and possibly it may be differentially modulated by GA. Therefore, we studied the effect of GA at the transcriptomic level by altering the GA homeostasis with exogenous application of GA_3_. This treatment was applied on the same date in both varieties in the 2017–2018 season, when they were at a similar physiological stage, as revealed by the IAD parameter (Fig. 3A,B), allowing the transcriptomic data to be comparable between varieties. The date selected for the treatment was 35 DAFB in the 2017–2018 season, five and 15 days before early- and mid-season variety color initiation, respectively, to characterize the molecular events modulated by GA at the onset of ripening and the early differences between both varieties.

To characterize the effect of GA_3_, we considered CT4-T0 and GT4-T0 comparisons, which contain GA-modulated genes since they exclude the genes that changed independently of the GA_3_ treatment (the intercepts of the diagrams; Fig. 4). As mentioned, CT4-T0 up and down comparisons contain genes that changed from T0 to T4 but did not change in the GT4-T0 comparison, i.e., GA-modulated genes since they no longer changed after the GA_3_ treatment. Therefore, it can be deduced that GA_3_ avoids their up or downregulation. The GT4-T0 up and down comparisons also contain GA-modulated genes, since their expression did not change from T0 to T4 in the control fruits, but the GA_3_ treatment up or downregulated them.

The GO category ‘response to hormone’ was overrepresented in the GT4-T0 comparison in the early-season variety (Table 2). This result was expected since GA_3_ treatment should produce an effect in the GA-pathway, which in turn interacts with several hormones routes in plants. The sensitivity to GA_3_ was revealed by the expression changes in GA pathway-related genes, where two genes encoding putative gibberellin 2-beta-dioxygenase 1 (GA2-ox) up-regulated (i.e. they were less repressed in the GT4-T0 comparison) in the early-season variety (Table 4). It is well accepted that GAs have negative feedback over their own biosynthesis, therefore these results are in agreement with this idea. Regarding the mid-season variety, only genes encoding gibberellin-regulated proteins were up-regulated (Table 4). Without any fold change filter, we detected a DELLA protein changing in both varieties and conditions, which was downregulated by GA_3_ in the early-season variety (1.97 in CT4-T0, 1.50 in GT4-T0) and strongly up-regulated in the mid-season variety (-1,67 in CT4-T0 1.76 in GT4-T0; not shown). DELLAs are often negative regulators of the GA response, though their expression changes are not always as relevant as the post-transcriptional regulation of these proteins.

The GO terms ‘protein phosphorylation’ and ‘photosynthesis’ were overrepresented in the CT4-T0 comparison of the early-season variety (Table 2), with genes downregulated in this comparison (Fig. 5A). This finding means that these genes downregulated from T0 to T4 in the control fruits, but they no longer changed after the GA_3_ application. Therefore, it is deduced that GA_3_ impedes this downregulation, which was expected since the photosynthetic rate and capacity are usually positively controlled by GAs^29,30^.

On the other hand, we observed that ‘auxin response’ was an overrepresented GO category in the GT4-T0 comparison in the early-season variety (Table 2). The genes in the overrepresented GO category ‘auxin response’ upregulated in the GT4-T0 comparison (Fig. 5A), and some of them were exclusive of the early-season variety (Fig. 7). Several putative genes of the Aux/IAA gene family coding for auxin-responsive proteins IAA29, IAA13, IAA6, among others, were upregulated from T0 to T4 (Table 4). These are mainly repressors of auxin responses in fruits^31^, therefore in the early-season variety, the auxin responses should turn off as ripening is initiating, since auxin usually antagonizes the ABA and ethylene promoting effect^6,7^. In this regard, auxin response should be activated by GA, since several genes encoding putative Aux/IAA repressors were downregulated in the GT4-T0 comparison. Reduced expression of these repressors should activate the auxin response; therefore, GA may be a positive regulator of the auxin pathway.

Interestingly, several genes encoding auxin-induced protein 15A and 15A-like were more upregulated in GT4-T0 comparison of the early-season variety than in the CT4-T0 comparison, whereas other different genes encoding a protein 15A-like were downregulated in the mid-season variety (Table 4). These results demonstrate that there is an antagonistic IAA response between both varieties. It also suggests a gene specialization, where only early-season auxin-induced protein 15A and 15A-like genes might be GA-responsive.

‘Carotene metabolic process’ GO category had two upregulated genes in the CT4-T0 comparison (Fig. 5A). Carotenoids are precursors of ABA, a key regulatory hormone that triggers ripening in non-climacteric fruits^2,32,33^. Several genes encoding putative ASR (abscisic acid stress ripening proteins) were upregulated only in the early-season variety. The *ASR* genes encode transcription factors induced by ABA and abiotic stress in several plant tissues, including fruits^34^. Additionally, two genes encoding an ABA degrading enzyme, abscisic acid 8’-hydroxylase 1 (CYP707A1), were more downregulated in the CT4-T0 than in the GT4-T0 comparison; hence, it is deduced that GA is a positive regulator of these genes. The silencing of *PavCYP707A2* in sweet cherry led to a delay in the transcript accumulation of several genes of the ABA and anthocyanin synthetic pathways^35^, supporting its role as a negative regulator of ripening. GA_3_ enhances its expression according to our results (Table 5), in agree to our recent report showing that GA_3_ increases the transcript abundance of *PavCYP707A2* five days after the treatment, but only in the early-season variety^17^.

In the mid-season variety, the GO term ‘carbohydrate metabolic process’ was overrepresented in both the GT4-T0 and the CT4-T0 comparisons (Table 2). Interestingly, the genes in this category were downregulated in the CT4-T0 comparison, whereas they were upregulated in the GT4-T0 comparison (Fig. 5B), implying that GA strongly activates these genes. GA_3_ strongly upregulated several genes encoding putative xyloglucan endotransglucosylase/hydrolases in the mid-season variety (data not shown). These enzymes are cell wall-modifying proteins and loosen cell walls hence conferring cell wall extensibility. Interestingly, transient size increase was observed only in the mid-season variety four days after the GA_3_ treatment (Fig. S5).

Another overrepresented GO term in the mid-season variety was ‘response to stress’ (Table 2). Genes in this category were downregulated in CT4-T0 but not in GT4-T0. Therefore, it is deduced that GA prevents their downregulation. A gene coding for a putative ethylene biosynthetic gene, 1-aminocyclopropane-1-carboxylate oxidase homolog 1-like, was also downregulated in CT4-T0 but not in GT4-T0 (Table 4). Possibly GA has a positive effect on ethylene synthesis, and this hormone, in turn, maintains some stress responses activated. Ethylene seems to play a promoting role in non-climacteric fruit ripening^36^; therefore, it is intriguing that GA, a hormone that antagonizes ripening, increases ethylene levels. These findings support the idea that GA, in late- or mid-season varieties, does not negatively affect ripening, and only produces a delay. Additionally, there is a more increased ethylene response in the mid-season variety; hence, the ethylene pathway might be significant in this variety.

The GO terms ‘regulation of transcription, DNA-templated’ and ‘regulation of nucleic acid-templated transcription’ were overrepresented in the mid-season variety, and they included genes that exclusively changed in this variety (Table 3; Fig. 7). Several genes coding for MYB44-like transcription factors were upregulated only in the mid-season variety (Table 5). Additionally, they were GA-downregulated as they were less upregulated in the GT4-T0 comparison. Also, a gene coding for an MYB44 transcription factor was present in both varieties, but it was more strongly expressed in the mid-season variety (Table 4). MYB44 proteins suppress the expression of *PP2C* genes encoding type 2C protein phosphatases^37^. PP2Cs are key proteins in the ABA regulatory network and are negative regulators of subfamily 2 of SNF1-related kinases (SnRK2s), therefore negatively regulating ABA responses^38^. *PavSnRK2s* and *PavPP2Cs* are expressed during sweet cherry fruit development^8^. Since *MYB44* related genes were upregulated in the mid-season variety, they could positively regulate the ABA response. Interestingly, these genes were less upregulated in the GT4-T0 comparison than in the CT4-T0 comparison, suggesting that GA downregulates the expression of *MYB44-*related genes. The expression variations in MYB44 related proteins may be a mechanism by which GA antagonizes the ABA pathway. MYB44 proteins transduce environmental signals and mediate stress responses^39^. They also mediate the interaction of hormone response pathways; for instance, MYB44 proteins regulate the antagonistic interaction between salicylic acid and jasmonic acid pathways^39^, and modulate the ABA signaling pathway. Therefore, it seems to be a crucial regulatory element for converging pathways.

### Ripening differences between maturity time contrasting varieties: What is in common and what is different?

We observed that some ripening-related molecular features were more activated in the early-season variety, including genes related to photosynthesis, carotenoid, and ABA pathway. Photosynthesis is a negative biomarker of ripening^40^, and in sweet cherry, chlorophyll decrease is one of the first events at the ripening initiation, even before sugar and anthocyanin content increase^33^. ‘Photosynthesis’ was an overrepresented category in the early-season variety (Table 2), and several photosynthesis-related genes were downregulated in the CT4-T0 comparison (Fig. 5A). These findings might be exclusive of the early-season phenotype, as this GO term was not overrepresented in the mid-season variety.

Auxin counteracts the ripening processes^2,6^. Our results show that a probable indole-3-pyruvate monooxygenase *YUCCA10* was upregulated in both varieties from T0 to CT4. This finding agrees with the increase in the IAA content detected in both varieties from 34 to 44 DAFB (Fig. S6A). Therefore, this hormone may prevent the ABA-induced ripening trigger from occurring too early. On the other hand, GA seems to control the IAA response pathway, as discussed above, rather than IAA biosynthesis, with differences between varieties in the expression of auxin-induced genes.

Carotenoid and ABA biosynthesis is a positive biomarker of ripening^40^. In the early-season variety, the ‘carotenoid metabolism’ category contained genes upregulated in the CT4-T0 comparison (Fig. 5A). Phytoene synthase and *NCED1* like genes were upregulated in this comparison in the early season variety (Table 4), possibly involved in carotenoid production and ABA biosynthesis. Additionally, in the CT4-T0 comparison, a probable carotenoid cleavage dioxygenase 4 chloroplastic gene was more downregulated in the early-season variety, which probably degrades carotenoids, since the putative orthologue of this gene in chrysanthemum (*Chrysanthemum morifolium*) contributes to white petal color^41^. Regarding ABA biosynthesis, *NCED1* transcript variations correlate with ABA increase during sweet cherry fruit ripening^4^. The *NCED1* upregulation in CT4-T0 in the early-season variety implies that ABA accumulates in this variety at the onset of ripening. We found that ABA content increases from 34 to 44 DAFB in the early-season variety, which occurred later in the mid-season variety (Fig. S6B). Interestingly, the mid-season variety with delayed color initiation also had delayed ABA accumulation, possibly due to a more expressed abscisic acid 8’-hydroxylase 4 gene in CT4-T0 than in the early-season variety.

Finally, phenylpropanoid early branches are negative biomarkers of non-climacteric ripening^40^. In this regard, we found that a tannin-related R2R3 MYB transcription factor was strongly repressed in the early-season variety, compared with the mid-season variety.

Chromosome reorganization, changes in histone methylation profile, and DNA hypomethylation are characteristic features of ripening initiation^42^. For instance, anthocyanin-deficient apple mutant had higher methylation levels in the promoter of a *MdMYB* gene^43^. Silencing of a methylase gene in the non-climacteric sweet pepper led to premature ripening^27^. We observed several downregulated genes in the CT4-T0 comparison encoding putative histone modification enzymes and DNA (cytosine-5)-methyltransferase 1-like (Table 5). These genes were downregulated in both varieties, suggesting that chromosome remodeling and DNA hypomethylation could be relevant at this stage of development. However, some were slightly more downregulated in the early-season varieties, suggesting that these processes could be advanced in this variety, leading to earlier ripening initiation in early-season varieties. Additionally, we identified some early-season variety genes, including histone-lysine N-methyltransferase *ATXR6*, involved in transcriptional repression in Arabidopsis^44^. On the other hand, some genes in the ‘DNA packaging’ GO categories were exclusive of the early-season variety (Table 3), suggesting that though DNA-related changes may occur in both varieties, they have variety-dependent particularities.

The GA pathway seemed to be downregulated earlier in the early-season variety since several gibberellin 2-beta-dioxygenase related genes were downregulated from T0 to T4 in the early-season variety (Table 4). These genes encode putative enzymes involved in GA degradation, previously characterized in grapevine fruits^9^. These genes downregulate with low GA content as they act in a negative feedback for controlling GA levels. Therefore, possibly a low GA content may be required for the onset of ripening to occur in the early-season variety, as it was observed (Fig. S1B and Fig. S1C).

GA_3_ treatment upregulates the DNA (cytosine-5)-methyltransferase in the early-season variety, so possibly higher methylation levels occur when GA content is elevated, thus impairing the ripening processes. The effect of GA_3_ on IAA and ABA pathways in the early-season variety, as discussed above, also supports that GA exerts a negative effect on the ripening processes.

Higher GA levels could be retarding the onset of ripening in the mid-season variety, possibly to reach full embryo development. The regulatory module controlling the timing of ripening initiation could involve GA repression of *MYB44-like* gene expression, a putative negative regulator of ABA signaling genes, *PP2Cs*. Less expression of *MYB44-like* genes could lead to the accumulation of negative *PP2Cs* transcripts. Therefore, more *PP2Cs* expression could imply that more ABA is needed to surpass this effect, explaining the ripening delay upon the GA_3_ treatment. In the mid-maturing sweet cherry variety Lapins, we previously found that GA_3_ increases *PavPP2C3* and *PavPP2C4* transcripts and delays the increase in the ripening index, IAD^17^, supporting the present findings. Contrary to GA_3_, we recently showed that ABA treatment advances the IAD increase related to *PavNCED1* transcript abundance accumulation and ABA content increase^45^. These results suggest a switch for the control of ripening timing where ABA advances and GA_3_ delays the ripening initiation in sweet cherry fruits.

**Figure 8:**
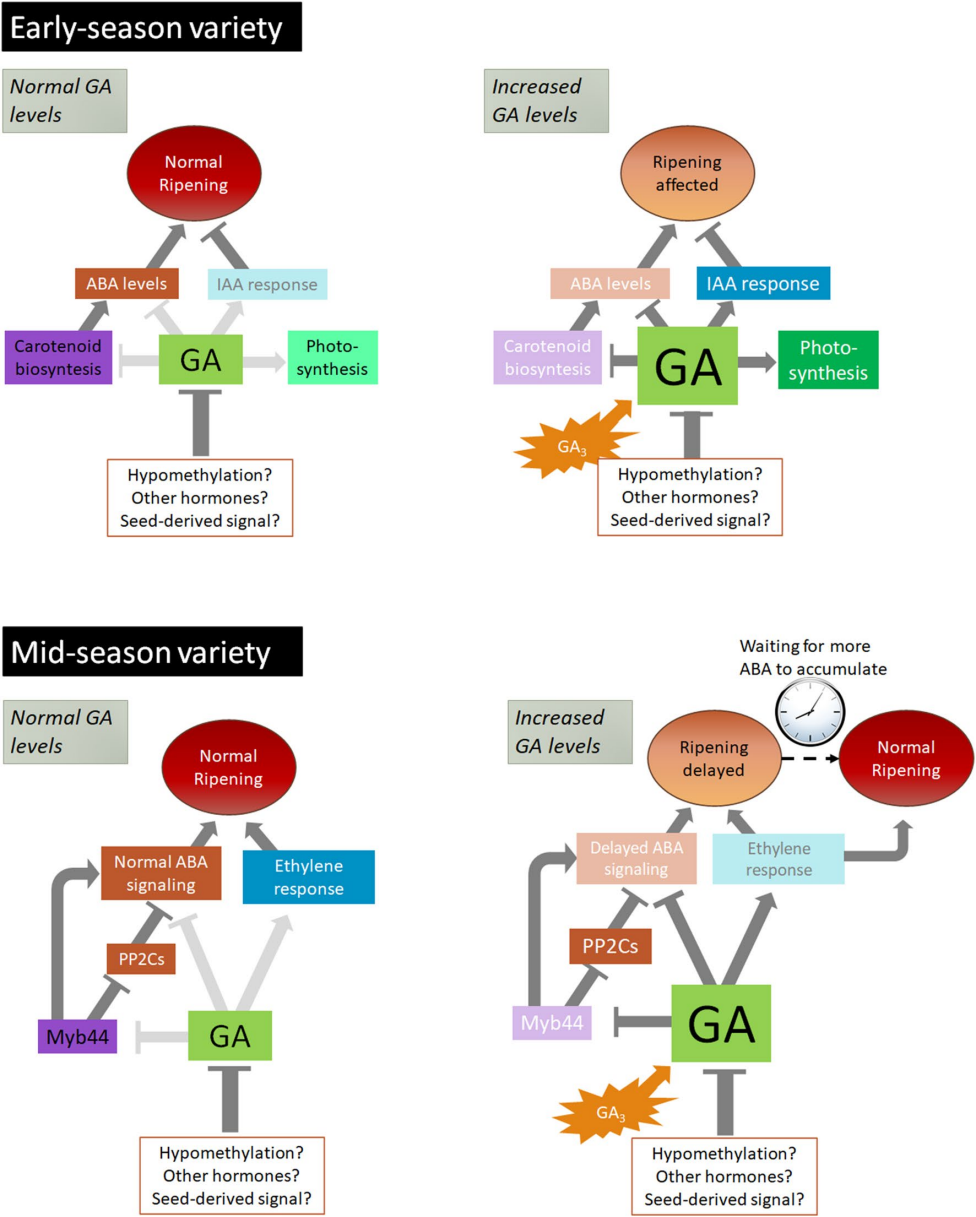
A working model for GA control of fruit ripening in sweet cherry. In early-season varieties, ABA pathways are activated earlier due to the downregulation of GA biosynthesis and negative regulation of GA over the ABA pathway. In contrast, in mid- or late-season varieties, ripening is delayed, in a mechanism possibly involving ABA negative regulators PP2Cs inactivation by MYB44. This effect is transient and can be overcame with higher ABA levels. In contrast, lower ABA in early-season varieties would be a more permanent effect of GA, potentiated by IAA pathway activation, which antagonizes ripening. Finally, GA should activate ethylene biosynthesis, which in turn controls the ABA pathway. Figure performed on Microsoft PowerPoint, version 18.2008.12711.0, https://www.microsoft.com/es-cl/microsoft-365/powerpoint.

The summary of possible molecular interactions controlling ripening in early- and mid-season varieties is depicted in our working model (Fig. 8). This work shows evidence supporting GA’s divergent role in the ripening process of two contrasting sweet cherry varieties and provides a better understanding of non-climacteric fruit ripening.

## Materials and methods

### Plant material

Sweet cherry (*Prunus avium*) fruits of four years old trees grafted on Cab-6P rootstocks growing in the *arboretum* of INIA Los Tilos Experimental Station, located in Buin, Región Metropolitana, Chile (33°42′S, 70°42′W), were selected for the experiments during the 2017–2018 and 2018–2019 seasons. The plants were in a 4 × 2 array, had regular nutritional, irrigation, and phytosanitary management, and did not receive plant growth regulator treatments. The varieties selected for the analyses were red varieties ‘Bing’ and ‘Celeste’, which have differences in the harvest time since ‘Bing’ is a mid-season variety, whereas ‘Celeste’ (‘Celeste, denomination Sumpaca, 13S.24.28, licensed to McGrath Nurseries Ltd in New Zealand) is an early-season variety^24^. These varieties’ phenology was determined every 2–4 days during both seasons based on fruit color variations from October in 2017 and 2018 (Tables S1 to S4). Three trees were used per variety for all the analyses and measurements, where each tree is a biological replicate. 0 DAFB (days after full bloom) was assigned when 50% of the flowers were open and was September 28 for both varieties in the 2017–2018 season, whereas in the 2018–2019 season, it was October 1 and 3 for early- and mid-season varieties, respectively.

### GA_3_ treatment and sampling

Three trees for each variety were used for GA_3_ treatments performed in 2017–2018 and 2018–2019. Six branches were selected from each tree, three control branches and three GA_3_-treated branches, following Usenik’s methodology^16^. GA_3_ (ProGibb 40% SG) was dissolved in water and applied to individual branches with a hand sprayer to run-off at a rate of 30 ppm when the fruits of each variety were in late Stage II of development, and fruit color transitioned from green to straw yellow^28^. Control branches were treated with water and protected from spraying with GA_3_, according to Usenik et al.^16^. The treatment was performed at the same hour of the day in both varieties and both seasons (12:00 GMT). Ten fruits per repeat (branch), i.e. 30 fruits per treatment, were sampled, with sample size according to Luo et al.^32^. Fruit samples were homogenous in color, size and form, and without any visible defect. The fruits were immediately frozen in liquid nitrogen after removal from the tree and stored at − 80 °C until used for RNA-seq analysis, anthocyanins estimations, and hormone quantification.

### Fruit parameters

For fruit width, a caliper was used to measure the equatorial diameter at the fruits’ widest point^14^. VIS/NIF device Cherry Meter (T.R. Turoni, Italy) was used to measure the ripening index, IAD (Index of Absorbance Difference), according to Nagpala et al.^25^. Non-destructive fruit width and IAD assessments were performed during the growing season until ripeness. Fruit width and IAD were measured individually in 20 fruits randomly selected from each tree.

Fruit weight, firmness, soluble solids content, and acidity (malic acid) were measured at the ripeness of each variety. Fruit weight was quantified using a portable mini scale, and firmness was determined on two opposite cheeks using a durometer device (Durofel T.R. Turoni, Italy), according to San Martino et al.^46^. Pocket Brix-Acidity Meter (PAL-BX|ACID3, ATAGO USA, Inc.) was used to quantify soluble solids content (SSC) and acidity as a percentage of malic acid, as reported by Sediqi et al.^47^. Color distribution was determined at harvest using a CTIFL color chart with 1 to 4 values (1 = light red, 2 = red, 3 = dark red, 4 = light mahogany). Fruit parameters were measured individually in 25 fruits randomly selected from each tree. For acidity and SSC, a subset of five fruits of the 25 fruits was measured, according to Chavoshi et al.^48^.

### RNA extraction and RNA seq-analysis

For RNA-seq analysis, samples were collected minutes before the GA_3_ treatment (T0 samples) and four days after the GA_3_ treatment (GT4 samples) or control treatment (CT4 samples) in the 2017–2018 season. RNA was extracted from 18 samples consisting of three replicates of T0, CT4, and GT4 samples (nine samples per variety). T0 samples of early- and mid-season varieties had a similar color and IAD value, which was around 0.4 in T0 samples in the 2017–2018 season. For the RNA-seq analysis, the 2017 samples were used (GA_3_ application on November 2, 2017; 35 DAFB, 12:00 GMT in both varieties).

Total RNA was isolated from 0.5 g of ground flesh- and peel-enriched tissue using the CTAB-method with minor modifications, according to Meisel et al.^49^. GeneJET RNA Cleanup and Concentration Micro Kit (Thermo Scientific, San Diego, CA, USA) were used for purifying the RNA samples. Purity values, A260/230 and A260/A280, were around 2.0 in all the samples.

For RNA-seq analysis of the 18 fruit samples, 1 µg of RNA with RIN (RNA integrity number) > 7.0 was used to generate cDNA libraries of the fruit samples using the Illumina TruSeq stranded mRNA Library Preparation Kit, according to manufacturer’s instructions. The libraries were sequenced in an Illumina platform (Illumina NovaSeq6000 at Macrogen Inc.), and 100 bp paired-end reads were generated. Data was generated using the base-calling software CASAVA v1.8.2 for forward and reverse segments.

### Analysis of differentially expressed genes

Adaptors, low-quality bases, and short sequences trimming were performed using CLC Genomics Workbench version 11.0.1 following parameters: Q ≥ 20; no more than two ambiguities; final read length ≥ 50 bp. Sequence mapping to the reference genome^50^ was performed using CLC Genomics Workbench version 11.0.1 with the following parameters: similarity = 0.9; length fraction = 0.6; insertion/deletion cost = 3; mismatch cost = 3, and unspecific match limit = 10. Expression levels were normalized by calculating RPKM (Reads Per Kilobase Million) value. Transcript abundances were fitted using a general linear model (GLM) and differential expression of treatments tested with the Wald test against control groups^51^. Differentially expressed genes (DEGs) were defined as having an absolute fold-change value of at least two between T4 and T0, with an adjusted *p-*value using a false discovery rate (FDR) with at least a 95% significance^52^.

Functional annotation of *P. avium* reference transcripts^50^ was performed by BLAST2GO software version 5.2.5^53^. First, a BLASTx search was performed against the NCBI NR database^54^ with an *e-*value cutoff of 1e-6 and HSP length cutoff of 33. Then, INTERPROSCAN analysis^55^ was performed with BLAST2GO default parameters. Finally, the data from BLAST searches, INTERPROSCAN terms, enzyme classification codes (EC), and metabolic pathways (KEGG, Kyoto Encyclopedia of Genes and Genomes) were merged in gene ontology (GO) terms for a comprehensive functional range cover in the functional annotation. The BLAST2GO program defaults were used in all mapping and annotation steps, and the false discovery rate (FDR) cutoff was set to a 0.05% probability level. GO over-representation analysis was performed with the Fisher’s Exact Test Enrichment Analysis using BLAST2GO tools and integrated FatiGO package^56^ with default parameters.

Venn diagrams were constructed using the online tool VENNY version 2.1^57^, whereas Heatmaps were constructed with the online tool Morpheus (https://software.broadinstitute.org/morpheus/).

### Hormone quantification

ABA, IAA, GA_1,_ and GA_4_ were measured in both varieties at 34, 38, and 44 DAFB in the 2018–2019 season. For the extraction, 10 mg of flesh- and peel-enriched tissue was freeze-dried, ground, and suspended in 80% methanol—1% acetic acid solution containing internal standards (deuterium-labeled hormones; OlChemim Ltd., Olomouc, Czech Republic). The mix was shaken for one hour at 4ºC, and the extracted fraction was maintained at − 20ºC overnight. The samples were centrifuged, and the supernatant was vacuum dried and then dissolved in 1% acetic acid. A reverse-phase column (OasisHLB) was used^58^, and the eluate was dried and dissolved in 5% acetonitrile—1% acetic acid. An autosampler and reverse-phase UHPLC chromatography column, 2.6 µm Accucore RP-MS, 100 mm × 2.1 mm (ThermoFisher Scientific, San Diego, CA, USA) were used. Then the hormones were separated using a gradient of acetonitrile (2%-55%) containing 0.05% acetic acid, at a rate of 400 µL/min over 22 min. ABA, IAA, GA_1,_ and GA_4_ were detected in a Q-Exactive mass spectrometer (Orbitrap detector; ThermoFisher Scientific; San Diego, CA, USA). Targeted Selected Ion Monitoring and Electrospray Ionization in the negative mode were used to detect the hormones^58^. The quantifications were performed using external calibration curves with the Xcalibur 4.0 and TraceFinder 4.1 SP1.

### Analysis of anthocyanins

Anthocyanins were measured in control- and GA_3_-treated fruits at the full ripeness of both varieties using three replicates per date in the 2017–2018 season. For anthocyanins extraction, 0.1 g of flesh- and peel-enriched tissue was freeze-dried and ground. The tissue was mixed with 80% methanol solution, sonicated, shaken for 20 min, and left overnight in the dark at 4ºC. The samples were centrifuged for 10 min at 4 °C and 4,000 rpm and the supernatant filtered using a 0.22 µm PFTE membrane. Anthocyanins were separated using a Waters Acquity HSS T3 column, 1.8 μm, 100 mm × 2.1 mm, in a UPLC-MS/MS Waters Acquity system (Milford, MA, USA), as reported by Arapitsas et al.^59^. The anthocyanins were detected in a mass spectrometry Waters Xevo TQMS instrument with an ESI source. For data processing, Mass Lynx Target Lynx Application Manager was used. Cyanidin-3-*O*-rutinoside was used to estimate anthocyanin since it represented more than 98% of the total anthocyanins in both varieties.

### Statistical analysis

One-way ANOVA analysis with Tukey’s post hoc test was used for assessing differences between control- and GA_3_-treated fruits in estimated anthocyanins and fruit parameters, where three replicates per date were used to establish the mean differences, using the InfoStat software^60^. The graphs were performed using GraphPad Prism version 6.04 for Windows, GraphPad Software, La Jolla California USA, www.graphpad.com.

## Supplementary Information

Supplementary Information.

## Data Availability

The raw reads sequences from this work were submitted to NCBI’s Sequence Read Archive through the BioProject ID: PRJNA683645.

## Abbreviations

ABA: Abscisic acid
DAFB: Days after full bloom
IAD: Index of absorbance difference
GA: Gibberellin
GA_1_: Gibberellin A1
GA_3_: Gibberellin A3
GA_4_: Gibberellin A4
GA_7_: Gibberellin A7

## Plant parts use statement

The use of plants parts in the present study complies with international, national and/or institutional guidelines.
