# Supplementary material for: RNAseq reveals different transcriptomic responses to GA3 in early and midseason varieties before ripening initiation in sweet cherry fruits
Source: Sci Rep. 2021 Jun 22;11:13075. doi: 10.1038/s41598-021-92080-8 (PMC8219793; doi:10.1038/s41598-021-92080-8)

**RNAseq reveals different transcriptomic responses to GA_3_ in early and midseason varieties before ripening initiation in sweet cherry fruits.**

Authors: Nathalie Kuhn^A*^, Jonathan Maldonado^B*^, Claudio Ponce^C^, Macarena Arellano^C^, Alson Time^C,G^, Salvatore Multari^D^, Stefan Martens^D^, Esther Carrera^E^, José Manuel Donoso^F^, Boris Sagredo^F^, and Lee A. Meisel^C**^

**Supplementary Tables**

**Table S1:** Fruit phenology of the early-season variety during 2017-2018 season. The date of the GA_3_ treatment is highlighted in gray.

| Days after full bloom (DAFB) | Date | Phenology |
| --- | --- | --- |
| 25 | October 22, 2017 | 90% green and 10% light green |
| 28 | October 26, 2017 | 50% green and 50% light green |
| 32 | October 30, 2017 | 5% straw yellow and 95% light green |
| 35 | November 2, 2017 | 75% straw yellow and 25% light green |
| 37 | November 4, 2017 | 90% straw yellow and 10% light green |
| 40 | November 6, 2017 | 1% straw yellow with pink blushes and 99% yellow |
| 43 | November 9, 2017 | 5% straw yellow with pink blushes and 95% yellow |
| 47 | November 14, 2017 | 10% straw yellow with pink blushes 90% yellow |
| 50 | November 17, 2017 | 15% straw yellow with pink blushes 55% pink and 30% red |
| 53 | November 20, 2017 | 20% pink, 40% pink with red blush and 40% red |
| 55 | November 22, 2017 | 10% pink and 90% red |

**Table S2:** Fruit phenology of the mid-season variety, during 2017-2018 season. The date of the GA_3_ treatment is highlighted in gray.

| Days after full bloom (DAFB) | Date | Phenology |
| --- | --- | --- |
| 25 | October 22, 2017 | 40% green and 60% light green |
| 28 | October 26, 2017 | 50% green and 50% light green |
| 32 | October 30, 2017 | 5% straw yellow and 95% light green |
| 35 | November 2, 2017 | 75% straw yellow and 25% light green |
| 39 | November 4, 2017 | 90% straw yellow and 10% light green |
| 43 | November 11, 2017 | 95% straw yellow and 5% light green |
| 47 | November 14, 2017 | 99% straw yellow and 1% light green |
| 50 | November 17, 2017 | 5% straw yellow with pink blushes and 95% yellow |
| 53 | November 20, 2017 | 45% straw yellow with pink blushes 45% pink and 10% red |
| 57 | November 24, 2017 | 5% pink with red blushes, 70% pink and 25% red |
| 61 | November 28, 2017 | 50% pink and 50% red |
| 64 | December 1, 2017 | 1% pink and 99% red |

**Table S3:** Fruit phenology of the early-season variety during 2018-2019 season. The date of the GA_3_ treatment is highlighted in gray.

| Days after full bloom (DAFB) | Date | Phenology |
| --- | --- | --- |
| 30 | October 31, 2018 | 5% straw yellow and 95% light green |
| 34 | November 4, 2018 | 75% straw yellow and 25% light green |
| 38 | November 8, 2018 | 1% straw yellow with pink blushes and 99% yellow |
| 42 | November 12, 2018 | 5% straw yellow with pink blushes and 95% yellow |
| 45 | November 15, 2018 | 10% straw yellow with pink blushes and 90% yellow |
| 49 | November 19, 2018 | 10% straw yellow with pink blushes 55% pink and 35% red |
| 53 | November 23, 2018 | 5% straw yellow with pink blushes 35% pink and 60% red |
| 56 | November 26, 2018 | 5% pink and 95% red |

**Table S4:** Fruit phenology of the mid-season variety during 2018-2019 season. The date of the GA_3_ treatment is highlighted in gray.

| Days after full bloom (DAFB) | Date | Phenology |
| --- | --- | --- |
| 28 | October 31, 2018 | 40% green and 60% light green |
| 30 | November 2, 2018 | 5% straw yellow and 95% light green |
| 34 | November 6, 2018 | 70% straw yellow and 30% light green |
| 38 | November 10, 2018 | 90% straw yellow and 10% light green |
| 42 | November 14, 2018 | 95% straw yellow and 5% light green |
| 45 | November 17, 2018 | 1% straw yellow with pink blushes and 99% yellow |
| 48 | November 20, 2018 | 5% straw yellow with pink blushes and 95% yellow |
| 51 | November 23, 2018 | 40% straw yellow with pink blushes 50% pink and 10% red |
| 53 | November 25, 2018 | 10% straw yellow with pink blushes 60% pink and 30% red |
| 57 | November 29, 2018 | 50% pink and 50% red |
| 61 | December 3, 2018 | 40% pink and 60% red |
| 64 | December 6, 2018 | 5% pink and 95% red |

**Table S5**: Ripening related parameters in control- and GA_3_-treated fruit samples at harvest in early- and mid-season varieties, during 2018-2019 season. d.u., durometer units; SSC, soluble solids content; M.A., malic acid.

| Season | Variety | Treatment | Weight (g) | Firmness (d.u.) | SSC (ºBrix) | Acidity (M.A. %) |
| --- | --- | --- | --- | --- | --- | --- |
| 2018-2019 | Early-season Celeste | Control | 9.71^a**^ | 58.41^a^ | 17.57^a^ | 2.56^a^ |
|  |  | GA_3_^*^ | 10.82^b^ | 63.76^b^ | 16.26^a^ | 2.46^a^ |
|  | Mid-season Bing | Control | 7.60^a^ | 70.80^a^ | 18.49^a^ | 3.00^a^ |
|  |  | GA_3_ | 7.98^a^ | 75.72^b^ | 16.86^b^ | 2.84^a^ |

*GA_3_ was applied as the commercial product ProGibb 40% SG to individual branches a rate of 30 ppm. GA_3_ treatment was at the light green-to-straw yellow transition of fruits at 34 DAFB in Celeste and Bing.

** For each ripening related parameter, the significance of variation between control- and GA_3_-treated fruits was tested by one-way ANOVA analysis with Tukey's *post hoc* test, whereby different letters are significantly different means (p < 0.05).

**Table S6**: Filtered and mapped reads, including average length and % of mapping in the samples of the early- and mid-season varieties.

|  |  |  | Filtered reads | | | mapped reads | |
| --- | --- | --- | --- | --- | --- | --- | --- |
| Sample_ID | Variety | Sample | number of reads | average length (bp) | total data (GB) | number of reads | mapped (%) |
| CT0_1 | **Early-season var., Celeste** | T0  (T0 control) | 40.194.252 | 98 | 3.91 | 27.185.190 | 68.1 |
| CT0_2 |  |  | 45.723.783 | 98 | 4.48 | 30.659.565 | 67.3 |
| CT0_3 |  |  | 39.549.454 | 98 | 3.85 | 26.967.053 | 68.7 |
| CCT4_1 |  | CT4  (T4 control) | 58.315.964 | 98 | 5.70 | 39.009.338 | 67.2 |
| CCT4_2 |  |  | 45.970.179 | 98 | 4.49 | 30.766.406 | 67.2 |
| CCT4_3 |  |  | 42.605.223 | 98 | 4.16 | 29.407.862 | 69.4 |
| CGT4_1 |  | GT4 (T4 GA_3)_ | 59.030.486 | 98 | 5.78 | 40.373.031 | 68.6 |
| CGT4_2 |  |  | 55.235.875 | 98 | 5.40 | 37.361.578 | 67.9 |
| CGT4_3 |  |  | 40.002.472 | 98 | 3.91 | 27.594.686 | 69.2 |
| BT0_1 | **Mid-season var., Bing** | T0  (T0 control) | 41.643.228 | 98 | 4.08 | 28.120.653 | 67.6 |
| BT0_3 |  |  | 39.679.885 | 98 | 3.87 | 27.180.317 | 68.9 |
| BT0_4 |  |  | 41.820.380 | 98 | 4.09 | 27.931.675 | 67.1 |
| BCT4_1 |  | CT4  (T4 Control) | 39.616.452 | 98 | 3.87 | 27.484.555 | 69.7 |
| BCT4_3 |  |  | 51.657.227 | 98 | 5.04 | 36.399.884 | 70.8 |
| BCT4_4 |  |  | 53.008.658 | 98 | 5.20 | 36.014.965 | 68.0 |
| BGT4_1 |  | GT4 (T4 GA_3)_ | 59.929.363 | 98 | 5.85 | 41.430.642 | 69.4 |
| BGT4_3 |  |  | 44.291.028 | 98 | 4.33 | 31.036.970 | 70.3 |
| BGT4_4 |  |  | 61.584.216 | 98 | 6.03 | 42.669.533 | 69.5 |
| Average |  |  | 47.769.896 | 98 | 4.67 | 32.644.106 | 68.6 |
| Total |  |  | 859.858.125 |  | 84.05 | 587.593.903 | 68.3 |

**Supplementary Figures**

**Figure S1.** Changes in fruit size and GA_4_ and GA_1_ content in early- and mid-season varieties in the 2018-2019 season. A, Fruit size as equatorial diameter of the fruits at different days after full bloom (DAFB). Bars represent the mean of three independent biological replicates ± SD. B, Endogenous GA_4_ content in the fruits on a dry weight (DW) basis at different DAFB. C, Endogenous GA_1_ content in the fruits on a dry weight (DW) basis at different DAFB. Bars represent the mean of three independent biological replicates ± SEM.

**Figure S2.** Effect of GA_3_ treatment on fruit color at ripeness in early- and mid-season varieties (56 and 59 DAFB, respectively) in the 2018-2019 season. GA_3_ was applied as the commercial product ProGibb 40% SG to individual branches at a rate of 30 ppm. GA_3_ treatment was applied at the light green-to-straw yellow transition of fruits at 34 DAFB of both varieties. A and B, Color distribution according to CTIFL color chart (1 is the lightest and 4 is darkest fruit color detected). The percentage (%) is calculated considering the number of fruits having a given category over the total number of fruits. C and D, Representative picture of control- and GA_3_-treated fruits.

**Figure S3.** Changes in IAD (Index of Absorbance Difference) in control- and GA_3_-treated fruits in the 2018-2019 season. A, IAD in early-season variety at different DAFB. B, IAD in mid-season variety at different DAFB. In A and B, bars represent the mean of three independent biological replicates ± SE. The significance of variation between control- and GA_3_-treated fruits at harvest of each variety was tested by one-way ANOVA analysis with Tukey's *post hoc* test, whereby an asterisk denotes significantly different means (p < 0.05).

**Figure S4.** Heat map showing the clustering of the 100 more expressed unigenes in the 18 sequence libraries.

**Figure S5.** Changes in fruit size in early- and mid-season varieties in the 2017-2018 season in response to GA_3_. Fruit size as equatorial diameter of the fruits at different days after full bloom (DAFB). Bars represent the mean of three independent biological replicates ± SD. GA_3_ treatment date indicated with an arrow.

**Figure S6.** Changes in ABA and IAA content in early- and mid-season varieties in the 2018-2019 season. A, Endogenous ABA content in the fruits on a dry weight (DW) basis at 34, 38 and 44 DAFB. B, Endogenous IAA content in the fruits on a dry weight (DW) basis at 34, 38 and 44 DAFB. Bars represent the mean of three independent biological replicates ± SEM.

Figure S1





Figure S2





Figure S3





Figure S4





Figure S5





Figure S6


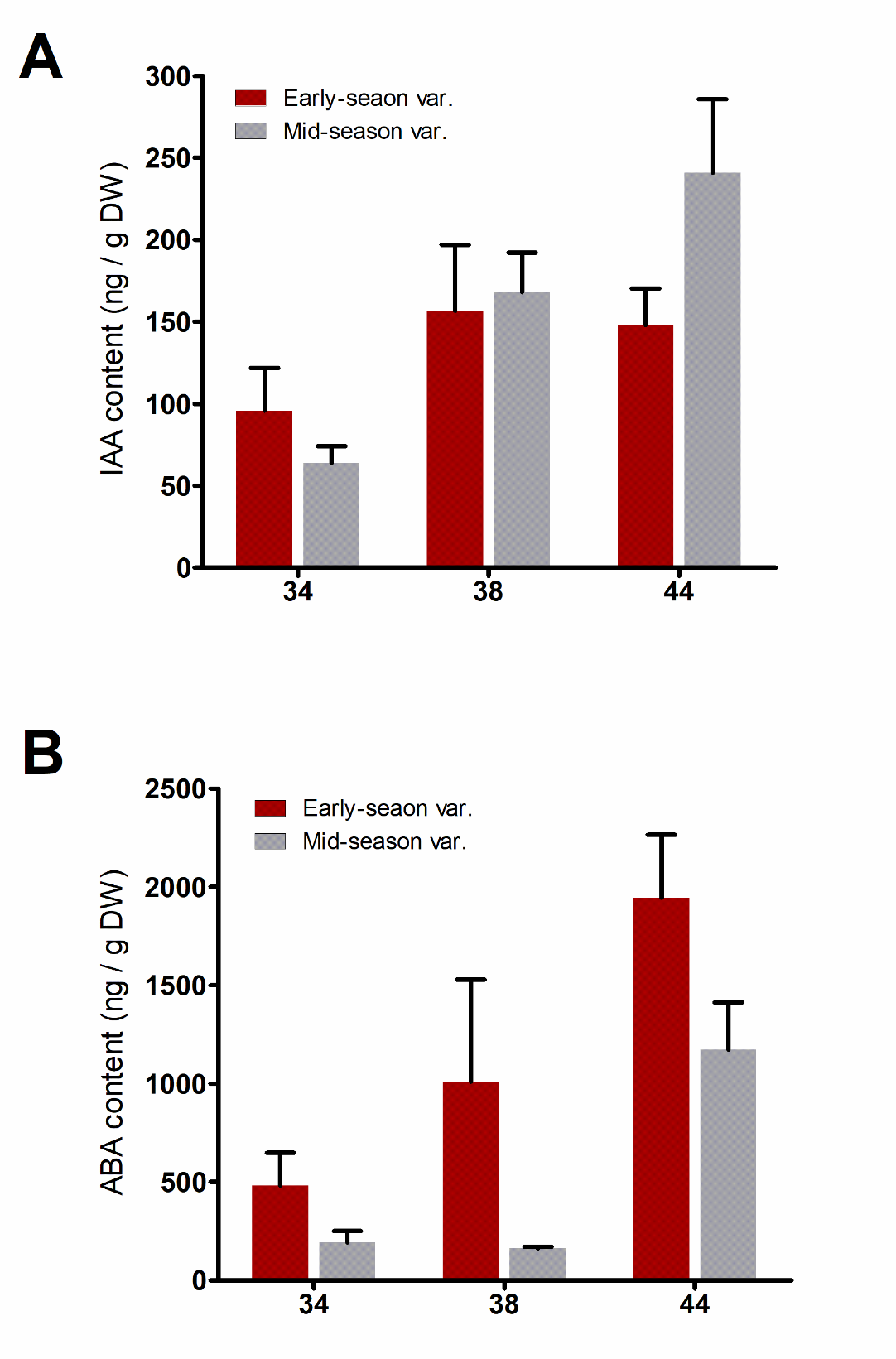

Supplement: Supplementary file 1 — Supplementary Information. [file 41598_2021_92080_MOESM1_ESM.docx]
